# Enterococcal surface protein Esp is not essential for cell adhesion and intestinal colonization of *Enterococcus faecium *in mice

**DOI:** 10.1186/1471-2180-9-19

**Published:** 2009-01-29

**Authors:** Esther Heikens, Masja Leendertse, Lucas M Wijnands, Miranda van Luit-Asbroek, Marc JM Bonten, Tom van der Poll, Rob JL Willems

**Affiliations:** 1 Department of Medical Microbiology, University Medical Center Utrecht, Heidelberglaan 100, Utrecht 3584 CX, the Netherlands; 2 Julius Center for Health Studies and Primary Care, University Medical Center Utrecht, Heidelberglaan 100, Utrecht 3584 CX, the Netherlands; 3 Center for Infection and Immunity Amsterdam (CINIMA), Academic Medical Center, Meibergdreef 9, Amsterdam, 1105 AZ, the Netherlands; 4 Center for Experimental and Molecular Medicine, Academic Medical Center, Meibergdreef 9, Amsterdam 1105 AZ, the Netherlands; 5 Laboratory for Zoonoses and Environmental Microbiology (LZO), National Institute for Public Health and the Environment (RIVM), Antonie van Leeuwenhoeklaan 9, Bilthoven 3721 MA, the Netherlands

## Abstract

**Background:**

*Enterococcus faecium *has globally emerged as a cause of hospital-acquired infections with high colonization rates in hospitalized patients. The enterococcal surface protein Esp, identified as a potential virulence factor, is specifically linked to nosocomial clonal lineages that are genetically distinct from indigenous *E. faecium *strains. To investigate whether Esp facilitates bacterial adherence and intestinal colonization of *E. faecium*, we used human colorectal adenocarcinoma cells (Caco-2 cells) and an experimental colonization model in mice.

**Results:**

No differences in adherence to Caco-2 cells were found between an Esp expressing strain of *E. faecium *(E1162) and its isogenic Esp-deficient mutant (E1162Δ*esp*). Mice, kept under ceftriaxone treatment, were inoculated orally with either E1162, E1162Δ*esp *or both strains simultaneously. Both E1162 and E1162Δ*esp *were able to colonize the murine intestines with high and comparable numbers. No differences were found in the contents of cecum and colon. Both E1162 and E1162Δ*esp *were able to translocate to the mesenteric lymph nodes.

**Conclusion:**

These results suggest that Esp is not essential for Caco-2 cell adherence and intestinal colonization or translocation of *E. faecium *in mice.

## Background

Enterococci are normal inhabitants of the human gastrointestinal (GI) tract, but have emerged as important nosocomial pathogens with high-level resistance to antibiotics, such as ampicillin, aminoglycosides, and vancomycin [1]. They can cause a wide spectrum of diseases, including bacteremia, peritonitis, surgical wound infections, urinary tract infections, endocarditis, and a variety of device-related infections [1-11]. The majority of the enterococcal infections are caused by *Enterococcus faecalis*. However, in parallel with the increase in nosocomial enterococcal infections, a partial replacement of *E. faecalis *by *Enterococcus faecium *has occurred in European and United States hospitals [12-14]http://www.earss.rivm.nl.

Molecular epidemiological studies indicated that *E. faecium *isolates responsible for the majority of nosocomial infections and hospital outbreaks are genetically distinct from indigenous intestinal isolates [15,16]. Recent studies revealed intestinal colonization rates with these hospital-acquired *E. faecium *as high as 40% in hospital wards, while colonization in healthy people appeared to be almost absent [13,15,16]. It is assumed that adherence to mucosal surfaces is a key process for bacteria to survive and colonize the GI tract. Intestinal colonization of nosocomial *E. faecium *strains is a first and key step that precedes clinical infection due to fecal contamination of catheters or wounds, and in the minority of infections, through bacterial translocation from the intestinal lumen to extraintestinal sites [17,18]. It is not known which factors facilitate intestinal colonization of nosocomial *E. faecium *strains. The enterococcal surface protein Esp, located on a putative pathogenicity island [19,20], is specifically enriched in hospital-acquired *E. faecium *and has been identified as a potential virulence gene. Esp is involved in biofilm formation [21] and its expression is affected by changes in environmental conditions, being highest in conditions that mimic the microenvironment of the human large intestines: 37°C and anaerobioses [22]. Furthermore, in one study, bloodstream isolates of *E. faecium *enriched with *esp *had increased adherence to human colorectal adenocarcinoma cells (Caco-2 cells) [23], suggesting a role of Esp in intestinal colonization. In contrast, adherence of *E. faecium *to Caco-2 cell lines was not associated with the presence of *esp *in another study [24]. In *E. faecalis*, Esp is also located on a pathogenicity island, although the genetic content and organization of the *E. faecium *and *E. faecalis *PAI is different. Esp of *E. faecalis *is also expressed on the surface of the bacterium [25,26] and is important in colonization of urinary tract epithelial cells [25]. By using a mouse model, Pultz et al. [27] showed that Esp does not facilitate intestinal colonization or translocation of *E. faecalis *in mice, however this does not automatically predict a lack function for *E. faecium *Esp in murine colonization. First data suggest that the function of Esp in both enterococcal species might be different. Esp of *E. faecium *is clearly involved in biofilm formation (see above) while there is controversy about the role of *E. faecalis *Esp in biofilm formation [28-31]. Furthermore, studies so far indicate that *E. faecalis *harbors more virulence determinants then *E. faecium*. For instance, besides Esp different determinants (GelE, BopD, *fsr *locus, and *bee *locus) are putatively involved in biofilm formation [32-34]. This suggests that virulence factors in *E. faecalis *play somewhat redundant or partially overlapping roles such that the absence of a single virulence factor, like Esp, has only minimal effect. To elucidate the role of Esp of *E. faecium *in bacterial adhesion and intestinal colonization, we studied an Esp mutant, constructed and described recently [21], and its Esp expressing parent strain for their ability to adhere to intestinal epithelial cells and intestinal colonization by using Caco-2 cells and a mouse model.

## Results

### Adherence assay to Caco-2 cells

**Figure 1:**
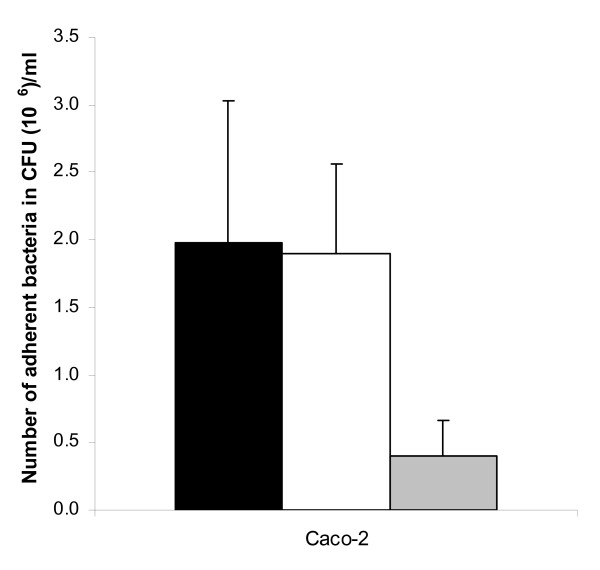
**Adherence to Caco-2 cells**. Adherence of E135 (grey bars), E1162 (black bars) and E1162Δ*esp *(white bars) to differentiated Caco-2 cells (14 days old). Adherence levels are expressed as the mean number of CFU per ml ± the standard deviation (SD).

To determine whether Esp contributes to adherence of intestinal epithelial cells, the Esp expressing *E. faecium *strain E1162, its isogenic Esp-deficient mutant (E1162Δ*esp*), and an *E. faecium esp*-negative strain (E135) were investigated for their ability to adhere to differentiated 14 days old Caco-2 cells. Strain E1162 exhibited high adherence to Caco-2 cells, while the *esp*-negative strain, E135, showed only low-level binding to Caco-2 cells (Figure 1). This difference in adherence was significant (P < 0.005). However, no significant difference in adherence to Caco-2 cells was observed between E1162 and E1162Δ*esp*.

### Intestinal colonization

To investigate the role of Esp in intestinal colonization and translocation to MLN, the Esp expressing E1162 and its isogenic Esp-deficient mutant (E1162Δ*esp*) were inoculated orally in mice separately or simultaneously in a mixed inoculum. Mice were kept under ceftriaxone treatment the entire experiment. Prior to any intervention no *E. faecium *was cultured from stools of mice. The mean enterococcal contents of the stool of naïve mice was 5 × 10^5 ^± 2 × 10^5 ^CFU/gram, these colonies were specified being *E. faecalis*.

**Figure 2:**
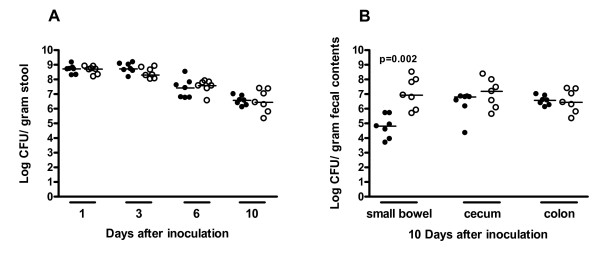
**Intestinal colonization**. Mice were orally inoculated with E1162 (black circles) or E1162Δ*esp *(open circles). (A) Numbers of E1162 and E1162Δ*esp *were determined in stool of mice at different time points after *E. faecium *inoculation. (B) After 10 days of colonization, numbers of E1162 and E1162Δ*esp *were determined in small bowel, cecum and colon. Data are expressed as CFU per gram of stool/fecal contents and medians are shown for 7 mice per group.

Both E1162 and E1162Δ*esp *were able to colonize the intestinal tract with comparable high numbers of cells for the entire 10 days of the experiment. One day after inoculation E1162 reached a median of 5.2 (range 2–15) × 10^8 ^CFU/gram of stool and E1162Δ*esp *of 5.1 (1.6 – 8.2) × 10^8 ^CFU/gram. Ten days after inoculation, the amount of both strains slightly reduced to 3.7 (1.3–10) × 10^6 ^and 2.7 (0.2–25) × 10^6 ^CFU/gram of stool, respectively (Figure 2A). Similar amounts of E1162 and E1162Δ*esp *were found in the stool of mice colonized when the mixed inoculum was administered (data not shown). After 10 days of colonization, all mice were sacrificed and *E. faecium *colonies obtained from small bowel, cecum, and colon contents were calculated. In both cecum and colon comparable amounts of E1162 (cecum contents 6.9 (0.04–7.3) × 10^6 ^and colon contents 3.9 (1.3–11) × 10^6 ^CFU/gram) and E1162Δ*esp *(cecum contents 10 (0.4–200) × 10^6 ^and colon contents 2.7 (0.2–24) × 10^6 ^CFU/gram) were isolated, from both separate (Figure 2B) and mixed inocula (data not shown). Significantly more E1162Δ*esp *(8.4 (0.5–300) × 10^6 ^CFU/gram) compared to E1162 (6.5 (0.5–52) × 10^4 ^CFU/gram) was isolated from the small bowel contents of mice when inoculated separately with E1162 wild type and the Esp-mutant strain (p = 0.002). This difference was not found in mice inoculated with the mixture of E1162 and E1162Δ*esp *(data not shown).

Both E1162 and E1162Δ*esp *were able to translocate to the MLN. From both of the separately inoculated groups of mice, three out of seven MLN were found positive for either E1162 or E1162Δ*esp*. No bacteria were cultured from blood. No pathological changes in the intestinal wall were observed in any of the colonized mice.

For both mono infection and mixed infection, randomly picked colonies were tested by MLVA to confirm strain identity. All colonies had the same MLVA profile belonging to *E. faecium *E1162(Δ*esp*).

## Discussion

Nosocomial *E. faecium *infections are primarily caused by specific hospital-selected clonal lineages, which are genetically distinct from the indigenous enterococcal flora. High rates of colonization of the GI tract of patients by these hospital-selected lineages upon hospitalization have been documented [13,15]. Once established in the GI tract these nosocomial strains can cause infections through bacterial translocation from the GI tract to extraintestinal sites [35,36]. The mechanism which promotes supplementation of the commensal enterococcal population by these nosocomial strains is not known. Destabilization of the GI tract through antibiotic therapy may provide nosocomial strains enhanced opportunities to gain a foothold in the GI tract. However, the effect of antibiotics is probably not the sole explanation for the emergence of nosocomial *E. faecium *infections since many antibiotics used in hospitals have relatively little enterococcal activity. This implicates that nosocomial *E. faecium *strains may possess traits that facilitate colonization of portions of the GI tract that the indigenous flora cannot effectively monopolize. Cell surface proteins like Esp, implicated in biofilm formation and specifically enriched in nosocomial strains, could represent one of these traits. Previously, it was shown that *E. faecium *is able to adhere to human and mouse intestinal mucus *in vitro *and becomes associated *in vivo *with the intestinal mucus layer of clindamycin treated mice [37-39]. This suggests an interaction between the bacterium and the mucus or with the epithelium itself. To examine the role of Esp in intestinal adherence and colonization, an Esp expressing strain of *E. faecium *(E1162) and its isogenic Esp-deficient mutant (E1162Δ*esp*) were studied for adherence to differentiated Caco-2 cells and colonization of murine intestines. E1162, a hospital-acquired strain, exhibited significantly higher adherence to Caco-2 cells than E135, a representative of the indigenous flora. These results are consistent with an earlier study performed by Lund et al. [23]. However, no difference in adherence to Caco-2 cells between the E1162 and the E1162Δ*esp *was found, indicating that Esp is not the determining factor responsible for the observed difference in Caco-2 cell adherence between nosocomial and indigenous *E. faecium *strains. This also implies that other determinants present in hospital-acquired *E. faecium *strains contribute to adhesion to intestinal epithelial cells. Comparative genomic hybridizations of 97 *E. faecium *nosocomial, commensal and animal isolates identified more than 100 genes that were enriched in nosocomial strains, including genes encoding putative adhesins, antibiotic resistance, IS elements, phage sequences, and novel metabolic pathways [40].

In addition, similar levels of intestinal colonization or translocation were found after inoculation with E1162 wild type or the isogenic Esp mutant E1162Δ*esp*. These data are in accordance with a study performed by Pultz et al. [27] in which they showed that Esp did not facilitate intestinal colonization or translocation of *E. faecalis *in clindamycin-treated mice. Only from the small bowel contents of mice when inoculated separately with E1162 wild type and the Esp-mutant strain significantly more E1162Δ*esp *compared to E1162 was isolated. This was an unexpected observation and we have no explanation for the fact that the levels of E1162Δ*esp *in the small bowel are as high as in the cecum. Relatively lower levels as seen for E1162 are more typical for the small bowel.

## Conclusion

Our data clearly demonstrate that Esp is not essential for high density colonization of the GI tract by nosocomial strains. Other possible candidate traits implicated in this process could include novel adhesins, like the novel cell surface proteins recently identified [41], bacteriocins, factors that resist specific or non-specific host defence mechanisms, and/or the ability to utilize new growth substrates. It is interesting in this respect that we recently identified a novel genomic island highly specific for nosocomial strains that tentatively encodes novel sugar uptake system [42].

For nosocomial *E. faecium *clones the GI tract serves as staging area from which they can disperse in and between patients, ultimately causing hospital-wide outbreaks. It is therefore of utmost importance to gain insight into the processes and determinants that promote intestinal colonization of nosocomial *E. faecium *strains. Only then we will be able to impede subsequent spread of these nosocomial clones.

## Methods

### Bacterial strains and growth conditions

In this study *E. faecium *strains E135, E1162 and E1162Δ*esp *were used. E135 is an *esp *negative community surveillance feces isolate, while strain E1162 is a hospital-acquired blood isolate, positive for Esp expression. The isogenic Esp-deficient mutant, E1162Δ*esp*, was previously constructed by introduction of a chloramphenicol resistance cassette (*cat*) resulting in an insertion-deletion mutation of the *esp *gene [21].*E. faecium *strains were grown in either Todd-Hewitt (TH) or Brain Heart Infusion (BHI) broth or on Tryptic Soy Agar (TSA) with 5% sheep red blood cells (Difco, Detroit, MI). Slanetz and Bartley (SB) agar plates were used to selectively grow enterococci. *E. faecium *strain E1162 and its isogenic mutant are high-level resistant to ceftriaxone (minimum inhibitory concentration > 32 μg/ml).

### Caco-2 cell cultures

Human colorectal adenocarcinoma cells, Caco-2 cells, were obtained from the American Type Culture Collection (HTB-37, ATCC, USA) and were cultured in Dulbecco's Modified Eagle Medium (DMEM; Gibco, Invitrogen, Paisley, UK) supplemented with 10% heat-inactivated fetal calf serum (Integro B.V, Zaandam, The Netherlands), 1% non-essential amino acids (Gibco), 2 mM glutamine (Gibco), and 50 μg/ml gentamicin (Gibco). Cells were collected every 7^th ^day by washing the monolayer twice with 0.022% disodium-ethylenediamine tetra acetic acid (di-Na-EDTA; Acros Organics, Morris Plains, NJ) in PBS and trypsinizing the cells using 50 μg/ml trypsine (Gibco), in 0.022% di-Na-EDTA in PBS. Cells were seeded at 1 × 10^6 ^cells in 10 ml DMEM in 75 cm^2 ^culture bottles (Costar, Corning, NY) and incubated in a humidified, 37°C incubator with 5% CO_2_. The culture medium was refreshed every 4^th ^day after passage of the cells. Differentiated Caco-2 cells were prepared by seeding cells from passage 25 to 45 in 12-wells tissue culture plates (Costar) at 1.6 × 10^5 ^cells/ml in DMEM. To each well 1 ml of this suspension was added and plates were incubated at 37°C with 5% CO_2 _for 14–16 days before use to allow the Caco-2 cells to differentiate. The medium in the wells was replaced by fresh medium three times a week.

### Adherence assay

Overnight-grown cultures of E135, E1162 and E1162Δ*esp *in BHI broth were diluted (1:50) and grown at 37°C to an OD_660 _of 0.4, while shaking. Bacteria were harvested by centrifugation (6,500 × g; 3 min) and resuspended in DMEM to a concentration of 1 × 10^7 ^CFU/ml. For each strain, 1 ml bacterial suspension was added to the wells (100 bacteria to 1 Caco-2 cell). Plates were centrifuged (175 × g; 1 min) and incubated for 1 h at 37°C in 5% CO_2 _to allow adherence to the Caco-2 cells. After incubation, monolayers were rinsed 3 times with DMEM and cells were lysed with 1% Triton X-100 (Merck, Darmstadt, Germany) in PBS for approximately 5 min at room temperature. Adherent bacteria were quantified by plating serial dilutions onto TSA plates and counting resultant colonies. Also the inoculum was plated to determine viable counts. The assay was performed simultaneously in 3 separate wells in duplicate and repeated on 3 different days.

### Mice

Specific pathogen-free 10-week-old female C57BL/6 mice (14 mice in total) were purchased from Harlan Sprague-Dawley (Horst, The Netherlands). The animals were housed in individual cages in rooms with a controlled temperature and a 12-h light-dark cycle. They were acclimatized for 1 week prior to usage, and received standard rodent chow and water ad libitum. The Animal Care and Use Committee of the University of Amsterdam approved all experiments.

### Induction of intestinal colonization

Mice were administered subcutaneous injections of ceftriaxone (Roche, Woerden, The Netherlands; 100 μl per injection, 12 mg/ml) 2 times a day, starting 2 days before inoculation of bacteria and continuing for the duration of the experiment. Two days after the initiation of the antibiotic treatment 2 × 10^9 ^CFU of E1162 or E1162Δ*esp *in 300 μl TH broth was inoculated by orogastric inoculation using an 18-gauge stainless animal feeding tube. In addition, in one experiment mice were administered a mixture of an equal amount (1.5 × 10^9 ^CFU) of E1162 and E1162Δ*esp *simultaneously. For all experiments, plate-grown bacteria were inoculated in TH broth and grown at 37°C to an OD_620 _1.0, while shaking. The inoculum was plated to determine viable counts. Mice were sacrificed after 10 days of colonization. Seven mice per group were examined.

### Collection of samples

Stool samples were collected from naive mice, 2 days after antibiotic treatment and 1, 3, 6 and 10 days after bacterial inoculation. Per mice, 2 stool pellets were collected, pooled, weighed (50–129 mg), and 1 ml of sterile saline was added. After 10 days of colonization mice were anesthetized with Hypnorm^® ^(Janssen Pharmaceutica, Beerse, Belgium; active ingredients fentanyl citrate and fluanisone) and midazolam (Roche, Meidrecht, The Netherlands), blood was drawn by cardiac puncture and transferred to heparin-gel vacutainer tubes. Mesenteric lymph nodes (MLN) were excised, weighed and collected in 4 volumes of sterile saline. Subsequently, the intestines were excised, opened and fecal contents of small bowel, cecum, and colon were weighed and 1 ml of sterile saline was added.

### Determination of bacterial outgrowth

The number of *E. faecium *CFU was determined in stool, MLN, blood, and fecal contents of small bowel, cecum, and colon. Stool, MLN, and fecal contents were homogenized at 4°C using a tissue homogenizer (Biospec Products, Bartlesville, UK). CFU were determined from serial dilutions of the homogenates and undiluted blood. Twenty μl of each dilution and 50 μl of undiluted blood, was plated onto SB agar plates and grown at 37°C for 44 h with 5% CO_2_. Colonies were counted, tested by PCR to confirm species identity, and corrected for the dilution factor to calculate CFU per gram of stool/MLN/fecal contents. MLVA was performed to confirm strain identity.

### PCR analysis to confirm species

Stool samples from naïve mice and from mice treated for 2 days with ceftriaxone were examined for presence of *E. faecium*. The lowest dilutions of stool homogenates that contained well-separated colonies were chosen and each colony of that dilution (12–24 CFU/20 μl diluted stool homogenate) was tested by PCR for presence of the housekeeping gene *ddl *(encoding D-alanine, D-alanine ligase) using the *E. faecium *specific primers ddlF (5'-GAG ACA TTG AAT ATG CCT) and ddlR (5'-AAA AAG AAA TCG CAC CG) [43]. The colonies were directly diluted in 25-μl-volumes with HotStarTaq Master Mix (QIAQEN Inc., Valencia, CA). PCR's were performed with a 9800 Fast Thermal Cycler (Applied Biosystems, Foster City, CA) and the PCR amplification conditions were as follows: initial denaturation at 95°C for 15 min, followed by 10 touchdown cycles starting at 94°C for 30 s, 60°C for 30 s, and 72°C (the time depended on the size of the PCR product) with the annealing temperature decreasing by 1°C per cycle, followed by 25 cycles with an annealing temperature of 52°C. All primers used in this study were purchased from Isogen Life Science (IJselstijn, The Netherlands).

For mono infection, colonies obtained from stool (1, 3, 6, and 10 days after bacterial inoculation), MLN, and fecal contents from small bowel, cecum, and colon were examined to confirm species identity. Colonies were randomly picked and presence of the *ddl *gene, in case E1162 was inoculated, or the *cat *gene, in case E1162Δ*esp *was inoculated, was assessed by PCR using primer pairs ddlF – ddlR and CmF (5'-GAA TGA CTT CAA AGA GTT TTA TG) – CmR (5'-AAA GCA TTT TCA GGT ATA GGT G) [21], respectively. When both strains were inoculated simultaneously, all colonies from the lowest dilution with well-separated colonies were picked (3–28 CFU/20 μl diluted homogenate). Species identity and the number of E1162 and E1162Δ*esp *were determined by multiplex PCR using primer pairs ddlF – ddlR and CmF – CmR. In PCR's, a colony of E1162 and E1162Δ*esp *was used as positive control and a colony of *E. faecalis *V583 [44] was used as negative control.

### MLVA to confirm strain identity

For both mono infection and mixed infection, colonies obtained from stool (1, 3, 6, and 10 days after bacterial inoculation), MLN, and fecal contents from small bowel, cecum, and colon were randomly picked and MLVA was performed to confirm strain identity. MLVA was performed as described previously [45].

### Histological examination

Small bowel, cecum and colon tissue were fixed in 4% buffered formalin and embedded in paraffin. Four-micrometer-thick sections were stained with hematoxylin-eosin and analyzed.

### Statistical analysis

Adherence data are expressed as the mean CFU per ml ± the standard deviation (SD). A two-tailed Student's *t *test was applied. Mouse colonization data are expressed as medians of CFU per gram of stool/fecal contents. Two group comparisons were done by Mann-Whitney *U *test. A *p*-value < 0.05 was considered statistically significant.

## Abbreviations

Esp: enterococcal surface protein; GI tract: gastrointestinal tract; Caco-2 cells: human colorectal adenocarcinoma cells; MLN: mesenteric lymph nodes; CFU: colony forming units; PCR: polymerase chain reaction; MLVA: Multiple-Locus Variable-Number Tandem Repeat Analysis.

## Authors' contributions

EH and ML carried out the design of the study, performed the mice and cell adherence experiments, and drafted the manuscript. LMW participated in the cell adherence experiments and helped to draft the manuscript. MvLA participated in the PCR analysis to confirm species. MJMB participated in the design of the study and helped to draft the manuscript. TvdP and RJLW participated in the design and coordination of the study, and helped to draft the manuscript. All authors read and approved the final manuscript.
